# Targeting Matrix Metalloproteinases and Their Inhibitors in Melanoma

**DOI:** 10.3390/ijms252413558

**Published:** 2024-12-18

**Authors:** Orest Szczygielski, Emilia Dąbrowska, Sylwia Niemyjska, Andrzej Przylipiak, Monika Zajkowska

**Affiliations:** 1 Clinic of Paediatric Surgery, Institute of Mother and Child, Kasprzaka Str 17a, 01-211 Warsaw, Poland; 2 General Hospital in Wysokie Mazowieckie, Szpitalna Str 5, 18-200 Wysokie Mazowieckie, Poland; 3 Department of Esthetic Medicine, Medical University of Bialystok, 15-267 Bialystok, Poland; 4 Department of Health Sciences, University of Lomza, 18-400 Lomza, Poland; 5 Faculty of Medicine with the Division of Dentistry and Division of Medical Education in English, Medical University of Bialystok, 15-269 Bialystok, Poland; monika.zajkowska@umb.edu.pl

**Keywords:** dermatological, skin disease, drugs, MMPs

## Abstract

Malignant melanoma is one of the most important dermatological neoplasms. The high mortality rate associated with this skin disease is primarily due to the occurrence of metastases, while the diagnosis and treatment of melanoma in its early stages has a favorable prognosis. Early detection is crucial because the success of treatment is directly related to the depth of cancerous growth. The family of matrix metalloproteinases (MMPs) plays a critical role in the initiation and progression of melanoma. Prominent MMPs, including MMP-1, MMP-2, MMP-3, MMP-9, MMP-13, and MMP-14, have been shown to significantly contribute to the development of melanoma. The tumor microenvironment, particularly the extracellular matrix (ECM), has emerged as a critical factor in modulating cancer progression. This review focuses on the role of matrix metalloproteinases and their inhibitors in ECM degradation and the subsequent progression of melanoma, as well as their potential as therapeutic targets.

## 1. Introduction

Melanoma is one of the less common neoplasms, accounting for fewer than five percent of all skin cancers. However, it is the most lethal, responsible for approximately 80% of skin tumor-related deaths worldwide [1]. Melanoma is a malignant neoplasm that originates from melanocytes, which are derived from neural crest cells and are responsible for the production of the pigment melanin. Melanocytes are predominantly located in the epidermis, hair follicles, along the surfaces of mucous membranes, within the meninges, and in the choroid layer of the eye [2,3,4]. Given the distribution of these cells, melanoma can arise in various anatomical sites, leading to classifications such as mucous membrane melanoma, ocular melanoma, and cutaneous melanoma. The most prevalent form is cutaneous melanoma, which accounts for over 90% of all melanoma cases, and its incidence is continually on the rise [5]. Cutaneous melanoma is the leading cause of mortality due to skin neoplasm, primarily attributable to its capacity for metastasis [6]. Consequently, the presence of metastatic disease is the most frequent cause of mortality associated with melanoma, and the estimated 5-year overall survival rate for patients with stage IV melanoma is approximately 23% [7]. Although contemporary treatment modalities (including surgical interventions, chemotherapy, targeted therapies, and immunotherapy) can delay tumor progression, patient prognosis remains unsatisfactory [8]. The therapies employed in the management of melanoma also present significant challenges, such as the occurrence of adverse effects, limited specificity for tumor cells, and diminished efficacy in the context of drug resistance [3]. Previous therapeutic approaches have not yielded satisfactory clinical outcomes. Thus, particularly in cases of metastatic melanoma, the identification of novel strategies to inhibit disease progression is essential for enhancing therapeutic efficacy [9].

The development and metastasis of cancer are facilitated by a complex interplay between malignant tumor cells and the surrounding non-malignant stroma, which comprises the extracellular matrix (ECM) and various stromal cells, including endothelial cells, fibroblasts, and infiltrating immune cells. Matrix metalloproteinases (MMPs) are zinc-dependent endopeptidases that play a critical role in ECM degradation and remodeling [10]. To date, 23 distinct MMPs have been identified in humans, which are classified broadly into collagenases (MMP-1, -8, -13, and -18), gelatinases (MMP-2 and -9), stromelysins (MMP-3, -10, -11), matrilysins (MMP-7 and -26), membrane-type MMPs (MMP-14, -15, -16, -17, -24, and -25), and other miscellaneous types based on substrate specificity or domain structure [11]. Excessive activation of MMPs can promote the development of characteristic features of cancer, such as increased inflammation, angiogenesis, and metastasis. Therefore, MMPs may serve as potential risk factors in the course of various cancers, including melanoma [12]. MMPs play a key role in tumor initiation, progression, and metastasis, and can influence the behavior of cancer cells by cleaving proapoptotic factors and generating their aggressive phenotype [13]. An emerging challenge in melanoma treatment is finding effective pharmacological and therapeutic methods for MMP suppression and targeted therapy [14].

The main aim of the current review was to present the current knowledge of the role of matrix metalloproteinases in the development of melanoma and the potential use of MMPs as therapeutic targets. The most relevant MMPs’ inhibitors (MMPIs) have been tested in clinical trials but the limitations of the use of MMPIs and recent efforts or advances in the development of highly selective MMP inhibitors are an important chapter of the current review.

## 2. Biology and Function of Matrix Metalloproteinases (MMPs)

Metalloproteinases are a group of proteolytic enzymes capable of digesting various components of the extracellular matrix. Despite their differences, they have been classified as one family due to similar structural characteristics. Most MMPs consist of five protein domains: a signal peptide, pro-peptide, catalytic domain, signal linker, and hemopexin domain [15,16]. The only exceptions are MMP-7 and -23, which lack a linker domain, and hemopexin [17]. The signal peptide is located at the amino terminus of the polypeptide chain and is responsible for directing the secretion of the enzyme from the surface of the endoplasmic reticulum toward the cell membrane. The pro-peptide domain contains a highly conserved Pro-Arg-Cys-Gly-(Val-/Asn-)Pro-Asp- motif in its structure, in which cysteine binds to a zinc ion and is responsible for maintaining the enzyme in an inactive form [10]. The catalytic domain is the next domain, responsible for the enzyme’s catalytic activity, with three histidines serving as the zinc-binding site. A signal linker is located between the catalytic domain and the carboxyl terminus of the protein, providing substrate specificity. The carboxyl terminus houses the last domain, which is structurally comparable to hemopexin. The hemopexin-like domain binds to matrix proteins and facilitates interactions between MMPs and tissue inhibitors of metalloproteinases (TIMPs) [18]. Some MMPs are distinguished from others by the presence of additional domains, such as a fibronectin-like domain containing three repeats of fibronectin type II motifs to which gelatin and type IV collagen substrates bind, an immunoglobulin-like domain rich in cysteine/proline, or a vitronectin-like domain containing repeats of a motif similar to the cell adhesion protein motif [19].

Matrix metalloproteinases (MMPs) are primarily produced by epidermal cells such as keratinocytes and fibroblasts, immunological cells such as neutrophils, macrophages, and dendritic cells, and tumor cells [20]. In humans, the MMP family comprises 24 proteins encoded by distinct genes; however, it is important to note that some MMPs, including MMP-4, MMP-5, and MMP-6, are not classified as human MMPs. These proteins are numbered from 1 to 28 and categorized based on their substrate specificity, which includes collagenases, gelatinases, matrilysins, stromelysins, membrane-type MMPs, metalloelastases, and other MMP types [21]. Collagenases are a specific group of MMPs capable of cleaving the primary fibrillar collagens (types I, II, and III) within their triple-helical structure [18]. Gelatinases, on the other hand, demonstrate proteolytic activity on denatured collagen, commonly referred to as gelatin. The substrates of stromelysins encompass a broad range of extracellular components, including proteoglycans, fibronectin, laminin, casein, gelatin, and collagen types III, IV, IX, and X. This category of MMPs also exhibits the capacity to activate other MMPs. Another significant group of MMPs is the matrilysins, which, unlike other MMPs, can exhibit intracellular activity. When activated within the cytoplasm, these enzymes may participate in the maintenance of the innate immune system by activating defensins, peptides known for their antibacterial properties [22]. Their extracellular functions extended to the hydrolysis of various matrix components, including fibronectin, collagen type IV, and proteoglycans [8]. The final major group of MMPs consists of membrane-type MMPs, which are situated on the external side of the cell membrane. Their substrates include collagen as well as other cell surface molecules such as fibronectin and laminin. Due to their localization on the cell surface, membrane-type MMPs are implicated in signaling processes and pathways that facilitate angiogenesis, cell migration, and various cellular functions, including proliferation, apoptosis, and differentiation [19]. Given their extensive substrate diversity, MMPs play significant roles in numerous biological processes related to extracellular matrix (ECM) remodeling. Consequently, alterations in the levels of MMP expression or activity are associated with a range of pathological processes [23,24].

The primary biological function of matrix metalloproteinases (MMPs) is the degradation of extracellular matrix (ECM) proteins and glycoproteins, as well as membrane receptors, cytokines, and growth factors [25,26]. The substrates of MMPs exhibit considerable variation: some are classified as components of the ECM, while others are constituents of the basement membrane [27]. Although each MMP possesses a unique set of substrates, there is a significant overlap in substrate specificity among these enzymes [28]. MMPs play a critical role in numerous physiological processes, including tissue repair and remodeling, cellular differentiation, embryogenesis, morphogenesis, cell proliferation and migration, angiogenesis, wound healing, and apoptosis. They are essential for maintaining a conducive extracellular environment and facilitating normal cellular interactions by preserving the structure of the ECM and regulating extracellular signaling pathways [29]. However, MMP-mediated ECM degradation can contribute to a variety of pathological conditions, including inflammatory and autoimmune disorders such as arthritis, neovascularization (neoangiogenesis), atherosclerosis, cardiovascular diseases, neurodegenerative disorders such as Alzheimer’s and Parkinson’s diseases, and various types of cancer [17].

Like all proteolytic enzymes, MMPs are secreted as proenzymes or zymogens, which remain inactive due to the interaction between the zinc ion in the catalytic domain and the sulfhydryl group of cysteine in the N-terminal domain [29]. The activation of MMPs requires the elimination of a mechanism referred to as the “cysteine switch”. Consequently, MMPs become partially activated and achieve full enzymatic activity through autocatalysis, in which the pro-domain is cleaved by the action of a proteinase. In the following stages, the enzyme may be progressively degraded and inactivated. MMP proteolytic activity is also regulated by particular protein inhibitors known as tissue inhibitors of metalloproteinases (TIMPs), which bind to MMP catalytic sites in a reversible manner. This family includes four proteins: TIMP-1, TIMP-2, TIMP-3, and TIMP-4. Furthermore, MMPs can also be inhibited by nonspecific inhibitors, such as α2-macroglobulin and thrombospondin-1 or -2 [30].

## 3. MMPs Endogenous Inhibitors

Maintaining a balance between the processes of extracellular matrix (ECM) formation and degradation is crucial for the homeostasis of the organism. A fundamental requirement for sustaining tissue stability is the presence of mechanisms that inhibit the activity of matrix metalloproteinases (MMPs), which is primarily mediated by tissue inhibitors of MMPs (TIMPs) [27]. TIMPs are endogenous proteins that selectively and reversibly bind to MMPs in a 1:1 stoichiometric ratio. Among these, TIMP-1, TIMP-2, and TIMP-4 are soluble secretory proteins, while TIMP-3 is an insoluble protein that binds to the ECM [31]. TIMP-1, which is secreted by most cells, was initially isolated from rabbit bone osteoclasts and is responsible for inhibition of the activity of all MMP types (binding exclusively strongly to MMP-9 and pro-MMP-9), except MMP-14, MMP-16, MMP-18, and MMP-19 [32].

TIMP-2, which was first identified from melanoma cells, is expressed in most tissues without being induced by growth factors [33]. This protein has a stronger inhibitory impact on MMP-2, whereas TIMP-3 is known as the only tissue inhibitor of all MMPs in mammals. TIMP-3 is expressed within tissues as a matrix protein and is present in the basement membranes of the eye and kidney cells, whereas TIMP-4 is predominantly expressed in the heart, ovary, kidney, colon, testis, brain, and adipose tissue cells [34,35]. Notably, TIMPs also interact with the zymogen forms of MMPs, although they do not exhibit inhibitory effects. Specifically, TIMP-1 and TIMP-3 interact with pro-MMP-9, while TIMP-2 and TIMP-4 interact with pro-MMP-2. In these interactions, only the C-terminus is involved, leaving the N-terminus available for binding to a secondary MMP molecule [36]. The expression of TIMPs within specific tissues can occur either constitutively or inducibly, depending on regulatory influences from cytokines, growth factors, and chemokines at the transcriptional level [34,35].

TIMP-1 and TIMP-2 exhibit a broad spectrum of inhibition across multiple MMPs, highlighting their significance as tissue inhibitors in human metabolism. Beyond their primary involvement in enzymatic inhibition, these proteins also exhibit antiapoptotic and antiangiogenic properties [37]. Notably, elevated expression of TIMP-2 has been observed in various neoplastic diseases, including lung cancer, prostate cancer, and melanoma [38,39,40]. Conversely, reduced expression of TIMP-2 is associated with tumor development and distant metastasis, while overexpression may yield the opposite effect, suggesting that it is not solely related to the inhibition of MMP activity. TIMP-1 and TIMP-2 exhibit antiangiogenic effects via inhibiting endothelial cell migration and proliferation, as well as extracellular protein aggregation. A distinct antiangiogenic effect has also been attributed to TIMP-3, which is linked to the mechanism of vascular endothelial growth factor (VEGF) blockade on endothelial cells [37]. Imbalances between MMP levels and their inhibitors contribute to the development of various cancer types. The degradation of the ECM is essential for malignant tumor formation, functioning as a critical component in tumor proliferation [25]. Dysregulation of TIMPs can alter important signaling pathways that influence cancer characteristics, such as the maintenance of proliferative signaling, evasion of growth suppression, and resistance to apoptosis, promoting replicative immortality [30].

## 4. MMPs and TIMPs in Melanoma

### MMPs and TIMPs in Melanoma Cell Migration, Tumor Growth, and Metastasis

**Figure 1:**
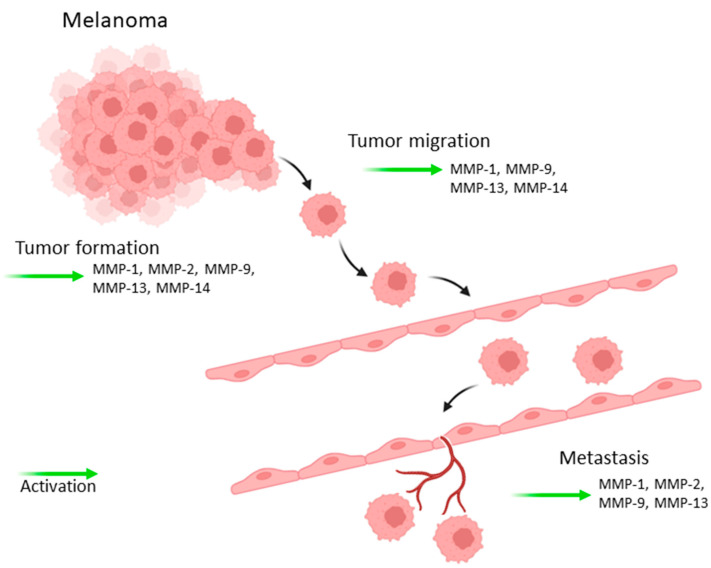
Roles of MMPs in tumor progression, invasion, and metastases.

Initially, matrix metalloproteinases (MMPs) were believed to facilitate metastasis primarily by degrading extracellular matrix (ECM) components and disrupting the basement membrane collagen. However, it is now recognized that MMPs play an important role in all stages of tumor development, influencing a variety of biological functions through the modulation of signaling pathways and the regulation of cytokines that are critical in immune response and tumor growth, particularly through the promotion of angiogenesis (Figure 1) [41,42].

The primary tumor secretes soluble factors including cytokines, chemokines, hormones, and metabolites, which affect both the future metastatic site and the bone marrow. This bone marrow remodeling produces VEGFR1 and VLA-4-expressing (VEGFR1+VLA-4+) progenitor cells, which are subsequently recruited to the premetastatic niche. The conditioning of this premetastatic niche enhances vascular permeability, induces thrombosis and inflammation, and activates stromal cells that collaborate in the remodeling of the ECM. The establishment of the metastatic niche is facilitated by bone marrow-derived cells (BMDCs) and type IV collagen found in the basement membrane [43,44,45]. In hypoxic conditions, type IV collagen is released within the tumor microenvironment and degraded by BMDCs with the involvement of MMP-2, contributing to tumor invasion and the distribution of metastases [46].

Tumor angiogenic processes are extensively regulated by interactions between tumor cells, endothelial cells, and the extracellular matrix. The hypoxia and nutritional deprivation that precede tumor growth, together with tumor vascularization processes, produce conditions that promote long-term tumor proliferation and tumor cells escape from the initial site to distant metastatic locations. MMPs released by tumor cells or tumor-associated stromal cells play an important role in neovascularization [47]. MMP-1, in particular, has been shown to promote angiogenesis and bone metastasis [48], whereas MMP-9, which is released by tumor-infiltrating neutrophils and macrophages, acts as an angiogenic factor, triggering the release of vascular endothelial growth factor (VEGF) and basic fibroblast growth factor (bFGF), both of which interact with the ECM [49,50].

To summarize, MMPs and tissue inhibitors of metalloproteinases (TIMPs) are involved not only in invasion and metastasis, but also in a variety of other important alterations required for tumor formation, such as proliferation, survival, angiogenesis, invasion, and migration [41]. The involvement of MMPs and TIMPs in the development of melanoma is discussed below.

## 5. Matrix Metalloproteinases: Characteristics and Roles in Malignant Melanoma Advancement

MMP-1, -2, -3, -9, -13, and -14 have been shown to play an important role in the development of melanoma [51,52]. Researchers have also found that altering MMP-9 and MMP-2 expression leads to increased activity and tumor aggressiveness. Both metalloproteinases have demonstrated potential predictive utility for distant metastases [53]. Table 1 shows the characteristics of key significant MMPs involved in the development of melanoma.

### 5.1. MMP-1, and MMP-13 (Collagenases)

Collagenases are the most prevalent extracellular matrix endopeptidases, with MMP-1 being the most common. Human MMP-1 is produced by a variety of cancer cells and surrounding stromal fibroblasts in response to transformed cell-derived stimuli. Collagens type I and III are key components of the extracellular matrix, and MMP-1 plays an important role in their degradation, thus participating in tumor progression [14]. Clinical research has indicated that MMP-1 is overexpressed in numerous malignancies, and its presence is associated with a poor prognosis, particularly in melanoma patients [52]. The participation of MMP-1 in tumor progression is largely associated with its ability to degrade fibrillar collagens, as well as with regulating the Th1/Th2 inflammatory response. Furthermore, MMP-1 and ADAMTS-1 proteolytically engage EGF-like growth factors in the osteolytic signaling cascade, promoting bone metastasis [54]. In a study by Wang et al. [55], MMP-1 expression was shown to be increased in uveal melanoma tissues compared to normal tissues, which was associated with shorter overall survival and disease-free survival. According to the researchers, MMP-1 expression levels could be applied as biomarkers to predict patient survival in uveal melanoma. In other studies, increased MMP-1 activity was shown to be correlated with increased invasiveness and metastasis in melanoma. It has also been proved that MMP-1 increased expression in invasive melanoma when compared to in situ melanomas [56]. Experimental studies in mice have revealed that reduced MMP-1 expression in a melanoma cell line results in a decreased ability of tumor cells to metastasize [42] and, on the contrary, its introduction into noninvasive melanoma cells induces a metastatic phenotype in vivo [43].

The role of matrix metalloproteinase-13 (MMP-13) in extracellular matrix (ECM) degradation is implicated in the degradation of collagen types I, II, III, and IV, as well as aggrecan, gelatin, and laminin-5 [14]. Conducted research indicates that MMP-13 may serve a dual function in melanoma, promoting both metastasis and the disruption of vasculogenic mimicry (VM) [57]. In human melanoma tissue samples, a positive correlation has been observed between MMP-13 expression and metastatic activity. This finding is corroborated by earlier studies demonstrating that MMP-13 enhances the invasiveness of cultured melanoma cells, aligning with evidence linking MMP-13 to melanoma progression and metastasis [58]. In research conducted by Zhao et al. [57], the mechanisms by which MMP-13 facilitates melanoma cell invasion and metastasis were further elucidated. It was determined that the proteolytic cleavage of laminin-5 by MMP-13 significantly contributed to increased invasiveness of the studied cells. Researchers hypothesize that the invasive and metastatic effects of MMP-13 may be mediated by the generation of smaller fragments of laminin-5 γ2, which could promote tumor cell invasion through the ECM.

Furthermore, macrophages have been identified as a source of MMP-13, utilizing this metalloproteinase to resorb the interstitial matrix. Additional cell types involved in the production of MMP-13 include neutrophils, which not only secrete MMP-9 and MMP-13 but also participate in the modulation of angiogenesis through ECM remodeling [57]. In the study by Zhao et al. [57], both macrophages and neutrophils were detected in the peritumoral stroma of melanoma biopsies, although MMP-13 production was observed in only a limited number of macrophages.

### 5.2. MMP-3 (Stromelysin)

MMP-3, a member of the stromelysin subfamily of matrix metalloproteinases, is primarily localized within the nucleus. However, it is important to note that, in certain contexts, MMP-3 may also be involved in transcriptional processes or apoptosis. This enzyme is produced by various cell types, including chondrocytes, endothelial cells, macrophages, and fibroblasts [59]. Research indicates that MMP-3 exhibits dual roles in cancer biology, possessing both tumor-promoting and tumor-suppressing properties that are contingent upon the specific substrates it interacts with. On one hand, MMP-3 exerts tumor-suppressive effects by catalyzing the production of factors that inhibit angiogenesis through the degradation of plasminogen and type VIII collagen. Conversely, its role in stimulating tumor cell proliferation is associated with the modulation of growth factors, such as transforming growth factor (TGF). Notably, the tumor-promoting functions of MMP-3 appear to be more prevalent than its tumor-suppressing capabilities. Consequently, MMP-3 is recognized as a potential prognostic factor in various cancer types [60]. For instance, there is evidence suggesting that MMP-3 plays a significant role in the development of the malignant phenotype of melanoma. This is particularly relevant in cases where elevated levels of MMP-3 are correlated with an increased incidence of experimental lung metastases in melanoma [61].

### 5.3. MMP-2 and MMP-9 (Gelatinases)

MMP-2 is primarily secreted by fibroblasts and other fibroblast-like stromal cells. This metalloproteinase plays a significant role in the promotion of cancer cell proliferation by contributing to the development of the tumor microenvironment. The underlying mechanism involves the degradation of adhesion molecules, including cadherins and integrins, along with cytoskeletal remodeling, which facilitates the detachment of cancer cells from the primary tumor tissue [62]. A crucial event during epithelial–mesenchymal transition (EMT) includes the MMP-mediated proteolytic cleavage of E-cadherin (CDH1), a key adhesion molecule, which results in reduced cell–cell adhesion. The loss of E-cadherin expression on cancer cell surfaces is thought to be a critical step in the EMT process [13]. The biological function of E-cadherin is to maintain skin integrity and inhibit the invasion of melanoma cells into the dermis by downregulation of adhesion receptors that are associated with invasion and the induction of apoptosis [63,64]. Simultaneously, there is an increased expression of N-cadherin (CDH2), a molecule associated with mesenchymal cells. The transformed cells migrate away from the primary tumor and penetrate the basement membrane, leading to the infiltration of adjacent tissues. This proposed mechanism is facilitated by the enzymatic activity of matrix metalloproteinases (MMPs), which support the migration of cancer cells [13]. Research conducted by Rotte et al. [65] showed that MMP-2 expression is significantly elevated in both primary and metastatic melanoma compared to normal and dysplastic lesions. Patients exhibiting high levels of MMP-2 expression experienced noticeably poorer survival outcomes compared to those with negative or moderate MMP-2 expression. Consequently, MMP-2 expression levels serve as a prognostic indicator for patient survival, irrespective of tumor size and ulceration [65].

Vascular endothelial growth factor (VEGF) is the only melanoma-derived molecule capable of directly activating endothelial cells in the absence of thrombin. According to recent studies, melanoma-derived VEGF binds the endothelial VEGF receptor 2 (VEGFR-2), resulting in rapid endothelial cell activation. These findings were supported by examination of tissue from patients with malignant melanoma, which revealed significant endothelial cell activation. Melanoma-derived MMP-2 regulates VEGF expression by activating integrin αVβ5 through an autocrine mechanism [66]. Tissue inhibitors of metalloproteinases (TIMPs) are responsible for prevention of MMP-dependent angiogenesis. TIMP-3 modulates angiogenesis by directly regulating VEGFR-2 activity and moderating the mitogenic effects of VEGF-A, whereas the actions of TIMP-2 are more complex, involving the inhibition of endothelial cell proliferation and migration [31].

MMP-9 plays a specific function in tumor cell invasion and the progression of primary tumors through its involvement in extracellular matrix (ECM) remodeling. Several cell types, including macrophages, neutrophils, fibroblasts, and endothelial cells have the capacity to synthesize and secrete MMP-9. This protease promotes the breakdown of basement membrane collagen, including type IV collagen [67]. Additionally, it is produced by both stromal and tumor cells, and its activity leads to the breakdown of ECM components (such as gelatin, type IV collagen, elastin, fibrillin, laminin, and fibronectin), thereby enhancing cellular mobility by compromising epithelial integrity. MMP-9, furthermore, has the ability to activate key growth factors, including transforming growth factor β (TGF-β), VEGF, and tumor necrosis factor α (TNF-α), thereby promoting tumor growth and angiogenesis [51,52]. Increased activity and potential predictive roles of MMP-2 and MMP-9 have been linked to the presence of distant metastases [53]. In a study by Manolescu et al. [52], the authors indicated a correlation between the duration period from the occurrence of the primary tumor to the emergence of metastases and the percentage of MMP-1- and MMP-9-positive cells (*p* = 0.0035; *p* = 0.0019, respectively) identified in metastatic lesions. Furthermore, it was noted that metastases characterized by a higher prevalence of MMP-1- and MMP-9-positive cells developed earlier than those with lower expression levels. These observations are further supported by literature revealing the increased aggressiveness of malignant melanocyte clones that are positive for MMP-1 and MMP-9 [14,68]

### 5.4. MMP-14 (Membrane-Type MMP)

MMP-14 plays a key role in enabling endothelial cells to invade and degrade surrounding tissue, facilitating the formation of new blood vessels [11]. This process is important in cancer development and progression. The presence of MMP-14 has been associated with adverse outcomes in patients with various types of cancer, including melanoma [69]. Melanoma cell invasion into the deeper layers of the skin and distant locations causes molecular alterations such as increased activation of tissue-degrading proteases, including MMPs. Research conducted to date shows that lymphatic endothelial cells interact with MMP-14 on the membranes of melanoma cells, facilitating cell–cell interactions in metastatic melanoma cell lines [70]. This process was accompanied by an MMP-14-dependent increase in Notch3 expression via an unknown mechanism. More recently, MMP14 has been found to bind with and activate Notch1 on the melanoma cell membrane, hence increasing cell proliferation [71]. Collagen type XIV has a crucial role in cell proliferation and migration. Addition of collagen XIV to the matrix deposited by cultured control fibroblasts led to decreased cell migration and adhesion [72]. In a mouse model, collagen XIV accumulation can be correlated with the loss of MMP14 from fibroblasts, resulting in decreased proteolytic processing. Collagen XIV has been identified as a novel substrate for MMP14. In human tissues, an inverse connection has been observed between collagen XIV and MMP-14. Interestingly, the increased expression of collagen XIV was observed around melanocytic nests of benign lesions with reduced MMP-14 expression, whereas in malignant melanomas collagen XIV was reduced and MMP-14 expression was increased. In accordance with these observations and the negative impact of collagen XIV in melanoma formation, MMP14 expression was established as low in benign lesions with a tendency to increase with the advancement of melanoma [73].

## 6. Pharmacological Targeting of Matrix Metalloproteinases and Their Tissue Inhibitors in Melanoma

The increased expression of matrix metalloproteinases (MMPs) and dysregulated levels of tissue inhibitors of metalloproteinases (TIMPs) significantly contribute to the dissemination of metastases and correlate with poor overall survival in melanoma patients. MMPs are considered potential therapeutic targets due to their association with unfavorable prognoses and early recurrences of melanoma. Inhibiting MMP activity during the early stages of tumorigenesis may prevent tumor growth. The identification of MMPs as key factors influencing tumor stage and patient prognosis has prompted the search for MMP inhibitors (MMPIs), which may represent an important milestone in cancer therapy [74].

**Figure 2:**
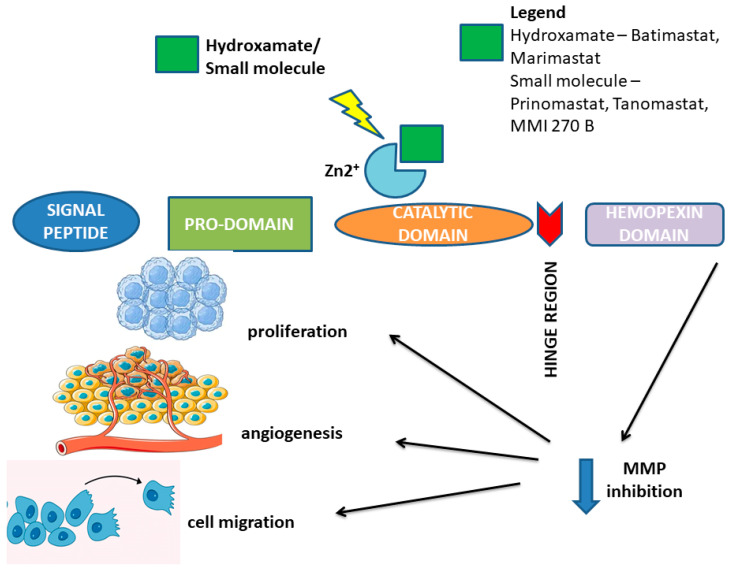
Schematic mechanism of action of various MMPIs in melanoma.

Matrix metalloproteinase inhibitors (MMPIs) represent a class of therapeutics specifically designed to inhibit the enzymatic activity of matrix metalloproteinases (MMPs). Their mechanisms of action in the context of melanoma can be delineated into several interrelated processes: inhibition of extracellular matrix (ECM) degradation, attenuation of tumor invasiveness, modulation of the tumor microenvironment, effects on cell signaling pathways, potentiation of immune responses, direct impact on melanoma cell viability, and potential for synergistic effects with other therapeutic modalities. By inhibiting MMPs, MMPIs effectively reduce the degradation of the extracellular matrix. This stabilization of the ECM can restrict the invasive potential of melanoma cells, thereby potentially mitigating metastasis. Furthermore, MMPIs can decrease the invasive characteristics of melanoma cells by obstructing MMP-mediated degradation of cell–ECM adhesion molecules (such as integrins) and components of the basement membrane. This action complicates the detachment and migration of cancer cells [74]. Additionally, MMPs play a pivotal role in the remodeling of the tumor microenvironment, influencing processes such as angiogenesis. Inhibition of MMPs has been associated with reduced angiogenesis, which may limit the supply of nutrients to the tumor and subsequently contribute to its growth inhibition. The impact of MMPIs on signaling pathways is also significant, as they can disrupt various signaling cascades modulated by MMPs—most notably those involved in tumor growth and metastasis, such as the PI3K/Akt and MAPK pathways—resulting in enhanced inhibition of cancer cell proliferation and survival. Moreover, the enhancement of immune responses may be attributed to the fact that tumors often exploit MMPs to escape immune surveillance. By inhibiting MMPs, MMPIs can bolster the immune system’s capacity to identify and eradicate melanoma cells, thereby potentially improving the overall immune response against the tumor [74,75]. Some MMPIs may also induce apoptosis in melanoma cells or compromise their proliferation independently of ECM interactions, further broadening their therapeutic utility. Additionally, MMPIs can be integrated with other therapeutic strategies, such as chemotherapy or immunotherapy, to augment treatment efficacy. By curtailing the metastatic potential of melanoma, MMPIs may enhance the effectiveness of these adjunctive treatments. Despite the recognized potential benefits of MMP inhibitors in melanoma therapy, their clinical efficacy has demonstrated variability, and the development of effective MMPIs has faced numerous challenges—including adverse side effects and insufficient specificity for distinct MMP isoforms. Ongoing research is essential to elucidate their precise role in melanoma treatment and to optimize their clinical application for improved patient outcomes [75]. A summary of the various mechanisms of MMPI action in melanoma is presented in Figure 2.

The rationale for employing MMPIs in treating patients has been supported by the fact that the expression and activity of numerous MMPs are markedly elevated in melanoma [74]. The compounds and functional groups used to inhibit the MMP activity are summarized in Table 2.

The first generation of synthetic MMPIs was based on hydroxamate chemistry, and included molecules like Batimastat, Marimastat, and Cipemastat. One of the first discoveries was Batimastat, a peptidomimetic chemical designed to mimic collagen, the most common MMP substrate. Batimastat revealed the ability to inhibit nearly all MMP family members, which is supported by preclinical findings indicating prospective anticancer activity. However, its development was impeded by insufficient water solubility, resulting in low oral bioavailability. Several phase I trials showed that Batimastat successfully suppressed tumor development and neoangiogenesis, especially in malignant melanoma liver metastases. Nonetheless, a variety of side effects were reported, including discomfort, fever, dyspnea, cough, nausea, peritonitis, and liver dysfunction [75,76]. These side effects eventually led to the discontinuation of further research on Batimastat, prompting the development of a more bioavailable medication, Marimastat [76].

Marimastat was designed as a next-generation oral analog, maintaining a similar mechanism of action to Batimastat, featuring a hydroxamate group that binds to and inhibits the catalytic zinc ion of various MMPs. Preclinical studies showed substantial potential, which facilitated its advancement to phase II and III clinical trials in metastatic cancer settings. Moreover, Marimastat was also better tolerated than Batimastat. Despite these advances, there have been reports of adverse effects related with a severe musculoskeletal condition that includes pain, inflammation, joint stiffness, muscle necrosis, gastrointestinal ulcers, and exhaustion [32]. This syndrome likely arose from Marimastat’s nonselective inhibition of MMPs as well as ADAMs (ADAM-10 and ADAM-17), which are involved in the cleavage of TNF-α. Inhibiting ADAM enzymes increased TNF-α levels, leading to increased inflammation [75,79]. Individuals treated with Marimastat have also developed significant fibrosis as a result of MMP-1 suppression. This inhibition impairs type I collagen interstitial remodeling, resulting in excessive extracellular matrix (ECM) deposition and fibrosis, which may have contributed to the severe side effects that caused Marimastat to be discontinued from usage [80].

Subsequent generations of inhibitors, such as Prinomastat, Tanomastat, and MMI 270 B, have enhanced selectivity and non-peptidomimetic characteristics. Prinomastat and Tanomastat exhibit inhibitory effects on MMP-2, MMP-3, MMP-9, MMP-13, and MMP-14. However, despite being characterized as more selective inhibitors and less frequently associated with musculoskeletal syndrome, they still resulted in various side effects, such as arthritis with swelling and pain, bone marrow suppression (including anemia and thrombocytopenia), gastrointestinal disturbances, and venous thromboembolism, particularly when combined with chemotherapy. These concerns, together with the absence of a significant tumor size decrease, resulted in discontinuation of these inhibitors in phase III clinical trials [14,75,77].

Attempts have also been made to develop highly selective MMP inhibitors. Efforts have focused on targeting the noncatalytic domains of MMPs, which differ among individual MMPs. Due to the fact that the active sites in the catalytic domain of MMPs are highly conserved, MMP inhibitors targeting the catalytic domains have the potential to target multiple MMPs rather than specific types of MMPs [81]. Studies have shown that MMPs play an important role in cancer progression and metastasis due to the presence of noncatalytic domains, such as the hemopexin (PEX) domain. Therefore, targeting the noncatalytic domain of MMPs may be a promising approach to develop specific MMP inhibitors [82,83].

Currently, monoclonal MMP-inhibiting antibodies are considered promising MMP-targeted therapies, as they exhibit higher target selectivity and superior pharmacokinetic profiles than small-molecule-based drugs. In preclinical experiments, highly potent antibodies against MMP-14 catalytic domain were found to be efficient in inhibiting MMP-14-mediated tumor development and metastasis, indicating that targeting MMP-14 is a viable therapeutic approach [84]. Antibodies targeting the PEX domain significantly decreased MMP14-mediated cell migration. Although larger proteins, such as monoclonal antibodies, have shown clinical value as tumor-targeting therapies, they are limited by their large molecular size, poor tumor penetration, and immunogenicity. Peptide ligands, which are smaller in size and immunogenic, may bypass these limitations. The corresponding peptides are highly selective for MMP-14’s PEX domain and do not interact with less conserved MMPs [83].

The introduction of monoclonal antibodies targeting inhibitory immune checkpoints has dramatically altered the treatment landscape for metastatic melanoma. The full activation of T lymphocytes is contingent upon two critical signals: the binding of major histocompatibility complex (MHC) I and II receptors on T lymphocytes to tumor-associated antigens (TAAs) presented by antigen-presenting cells (APCs) and the interaction of the CD28 receptor on T lymphocytes with CD80 and CD86 on APCs [85]. These interactions lead to T cell proliferation and cytokine release, culminating in an enhanced immune response. In the context of T cell activation, cytotoxic T lymphocyte antigen-4 (CTLA-4) serves as a competitive inhibitor, binding to CD80 and CD86 on APCs and thereby upregulating its own expression [86]. A diminished immune response to TAAs may also result from higher affinity binding of CTLA-4 and subsequent downregulation. The blockade of CTLA-4 signaling promotes T cell activation and proliferation, thereby enhancing T cell-mediated immunity and improving the efficacy of the patient’s immune response [85]. Ipilimumab, the first monoclonal antibody targeting CTLA-4 [87], has been the subject of extensive research involving 2901 patients across 25 clinical trials, demonstrating durable responses and prolonged survival in advanced melanoma cases. Current investigations are focusing on the combination of Ipilimumab with other therapeutic agents and assessing survival rates following treatment in previously treated patients. Initially receiving FDA approval in March 2011 for malignant melanoma, inclusive of unresectable or metastatic forms [88], ipilimumab has subsequently been approved for use in several other malignancies, such as renal cell carcinoma, colorectal cancer, and hepatocellular carcinoma [89,90]. Notably, it has produced significant improvements in durable response rates among patients and has paved the way for the development of subsequent classes of monoclonal antibodies. While effective immunotherapy with anti-CTLA-4 antibodies can be achieved, appropriate combination therapies are essential. Monotherapy with anti-CTLA-4 antibodies appears inadequate for the treatment of metastatic malignancies, likely due to the influence of the tumor microenvironment (TME). The TME is implicated in regulating tumor immune escape and mediating the drug sensitivity of tumor cells, suggesting that targeting the TME may represent a novel therapeutic strategy for malignancies. Matrix metalloproteinases (MMPs) are significant contributors to TME regulation, as they degrade the extracellular matrix and promote tumor angiogenesis. Additionally, MMPs are critical in facilitating the initiation and progression of cancer [91,92]. Jensen et al. [93] investigated the association between serum collagen biomarkers, vimentin turnover, and clinical outcomes in patients with metastatic melanoma receiving treatment with the anti-CTLA-4 antibody Ipilimumab. Their quantitative assessment of serum extracellular matrix (ECM) and tissue remodeling biomarkers prior to treatment revealed a correlation with treatment response and survival outcomes. High baseline levels of collagen biomarkers, including PRO-C3, C1M, C3M, and C4M, were associated with a poor response to Ipilimumab. Moreover, elevated levels of PRO-C3 and C4M correlated with reduced overall survival in patients. These biomarkers reflect ECM alterations and hold promise as potential prognostic indicators; however, further studies are warranted to evaluate their utility in the context of immunotherapy.

Nivolumab and Pembrolizumab, a class of anti-PD-1 antibodies approved three years after Ipilimumab, had high response rates, low relapse rates, and a well-controlled safety profile. Antibodies against PD-1 receptor ligands were subsequently developed to overcome the adverse effects of anti-PD-1 antibodies, and the combination of monoclonal antibodies (Ipilimumab plus Nivolumab) was demonstrated to increase response rates [94]. Specific immunological responses, such as those involving PD-1 receptor-positive T cells, have an impact on the progression of neoplastic disorders. Cancer cells with the PD-L-1 ligand on their surface attach to the PD-1 receptor on T lymphocytes, inhibiting their immune capabilities.

Monoclonal antibodies, by inhibiting PD-1, prevent the ligand from binding to the receptor, restoring T cell antitumor activity. Preliminary analyses of a phase III study showed significantly higher response rates and longer progression-free survival and overall survival with Nivolumab plus Ipilimumab or Nivolumab monotherapy than with Ipilimumab monotherapy in patients with advanced melanoma [95,96,97]. Nivolumab and Ipilimumab combined therapy was also clinically successful in individuals with metastatic melanoma and untreated brain metastases [98,99,100]. One aspect of the combination therapy with Nivolumab and Ipilimumab is that some patients who received it terminated treatment without receiving further systemic treatment for melanoma [97,101,102].

Wei et al. [103] developed a recombinantly engineered PD-1-derived fusion protein, dFv-ePD1, which comprises bivalent variable fragments (dFv) targeting MMP-2/MMP-9 in conjunction with PD-1. This construct presents opportunities for employing anti-gelatinase agents as targeted drug carriers. Single-chain variable fragments (scFv), owing to their moderate molecular size and rapid tissue penetration capabilities, are frequently utilized as optimal formats for drug delivery to cancer cells [104]. The bivalent variable fragment (dFv) from 3G11 demonstrated superior targeting and tumor-suppressive effects when compared to the monovalent scFv format [105]. Elevated levels of MMP-2/-9 expression correlate strongly with tumorigenesis, progression, invasion, metastasis, and poor prognostic outcomes, thereby making targeted inhibition of these matrix metalloproteinases a promising strategy for the treatment of metastatic tumors [106]. The dFv-ePD1 fusion protein demonstrated robust binding affinity to B16-F1 cells and human melanoma tissue microarrays through interactions with gelatinases, and it also exhibited binding capability to PD-L1. Moreover, dFv-ePD1 showed enhanced accumulation within tumor tissues compared to dFv alone. Researchers hypothesize that ePD1 may further facilitate tumor targeting due to its affinity for PD-L1, which is overexpressed in melanoma [103]. Current findings indicate that the dFv-ePD1 fusion protein retains the functional activity of dFv, likely leading to the inhibition of gelatinase activity [103]. Additionally, the dFv-ePD1 fusion protein exhibited a more pronounced inhibitory effect on the migration and invasion of B16-F1 cells relative to dFv. Prior investigations have identified a potential role for matrix metalloproteinases in the regulation of PD-L1 surface expression [107]. For instance, Zhao et al. [108] employed a pharmacological inhibitor to elucidate the role of the MMP-9 enzyme in modulating PD-L1 surface expression on BrafV600EPten−/− melanoma cells. Increasing concentrations of the MMP-9 inhibitor effectively abrogated the suppressive influence of melanoma-associated fibroblasts (MAFs) on PD-L1 expression in melanoma cells. These findings suggest that MAF-expressed MMP-9 plays a crucial role in shaping the therapeutic response to anti-PD-1 therapies. The introduction of anti-CTLA-4 and anti-PD-1 checkpoint inhibitors has significantly improved overall survival for patients with advanced melanoma.

Clinical trials are now taking place for monoclonal antibodies against MMP-9 (Andecaliximab, AB0041, AB0046, GS-5745), as well as MMP-1, MMP-2, MMP-3, and MMP-14. Among them, monoclonal antibodies against MMP-14 have proven effective in preclinical studies of melanoma [75,77]. Other more promising substances are also being investigated, such as MMP mRNA inhibitors, which have shown encouraging preliminary results in melanoma tests and may help prevent metastases. Nabipoorashrafi et al. [78] found a relationship between miR-143 and its unique target genes, which included MMP-9, epithelial cadherin (E-cadherin), vimentin, and CXCR4 (C-X-C chemokine receptor type 4), at both the mRNA and protein levels. Furthermore, a Western blot assay indicated that target gene expression was lowered at the protein level. The authors anticipated that miR-143 could decrease melanoma cell metastasis by targeting the aforementioned target genes.

## 7. Conclusions

MMPs, a family of zinc-dependent proteases, are involved in the remodeling and degradation of the extracellular matrix (ECM), playing an important role in metastasis. MMPs are integral to the inflammatory response and have been linked to the development of various malignancies and the progression of cancer, including melanoma. Additionally, MMPs have significant clinical relevance due to their potential as biomarkers. Although inhibiting MMP activity poses challenges for therapeutic applications, substantial research is underway to identify the most effective strategies for targeting MMPs in melanoma treatment. This review emphasizes the importance of investigating different types of MMPs to better understand their role in neoplasm progression and their potential as therapeutic targets in antitumor therapies.

## Figures and Tables

**Table 1:** Classification of human metalloproteinases (MMPs), expression and their functions in relation to melanoma.

Subgroup	MMP	Main Function	Role in Carcinogenesis	Expression in Melanoma
Collagenases	MMP-1	Collagen type I and III degradation	Facilitate tumor invasion in melanoma	↑
	MMP-13	Collagen type I, II, III, and IV degradation	Involved in invasive phenotype of melanoma	↑
Gelatinases	MMP-2	Collagen type IV degradation	Metastasis in melanoma	↑
	MMP-9	Degrade collage type IV	Tumor angiogenesis in melanoma	↑
Stromelysins	MMP-3	Collagen type I degradation Activate MMP-1, MMP-9	Activate pro-MMPs in melanoma	↑
Membrane type MMPs	MMP-14	Collagen type I, II, and III degradation	Tumor invasion Activation of MMP-2 Tumor angiogenesis in melanoma	↑

↑—overexpression.

**Table 2:** Activities of synthetic MMP inhibitor compounds.

MMPI	Activity	Source
Batimastat	Binds to and inhibits the catalytic zinc ion of various MMPs	[76]
Marimastat	Binds to and inhibits the catalytic zinc ion of various MMPs	[75]
Prinomastat	Selectively inhibits MMP-2, MMP-3, MMP-9, MMP-13, and MMP-14	[77]
Tanomastat	Binds to zinc and selectively inhibits MMP-2, MMP-3, MMP-9, MMP-13, and MMP-14	[77]
Andekaliksymab	Monoclonal antibodies against MMP-1, MMP-2, MMP-9, MMP-3, and MMP-14	[77]
miR-143	Decrease melanoma cell metastasis by targeting MMP-9	[78]

## Data Availability

No new data were created or analyzed in this study.
